# Tyrosine kinase inhibitor neratinib attenuates liver fibrosis by targeting activated hepatic stellate cells

**DOI:** 10.1038/s41598-020-71688-2

**Published:** 2020-09-08

**Authors:** Yong Joo Park, Hyoung-Tae An, Jong-Sung Park, Ogyi Park, Alexander J. Duh, Kwangmeyung Kim, Kyu Hyuck Chung, Kang Choon Lee, Yumin Oh, Seulki Lee

**Affiliations:** 1 Russell H. Morgan Department of Radiology and Radiological Science, Johns Hopkins University School of Medicine, Baltimore, MD 21205 USA; 2 Center for Nanomedicine At the Wilmer Eye Institute, Johns Hopkins University School of Medicine, Baltimore, MD 21205 USA; 3 Center for Theragnosis, Korea Institute of Science and Technology, Seoul, Korea; 4 School of Pharmacy, Sungkyunkwan University, Suwon, 16419 Korea

**Keywords:** Drug discovery, Molecular biology, Gastroenterology

## Abstract

Liver fibrosis, a common outcome of chronic liver disease characterized by excessive accumulation of extracellular matrix (ECM), is a leading cause of mortality worldwide. The tyrosine kinase inhibitor neratinib is a human epidermal growth factor receptor 2 (HER2) inhibitor approved by the FDA for HER2-positive breast cancer treatment; however, it has not yet been evaluated for liver fibrosis treatment. We elucidated the anti-fibrotic effects of neratinib in hepatic stellate cells (HSCs) and in vivo models of CCl_4_-induced liver fibrosis. HSC activation is a key step in liver fibrogenesis and has a crucial role in collagen deposition, as it is primarily responsible for excessive ECM production. The effect of neratinib on HSC was evaluated in transforming growth factor (TGF-β)-incubated LX-2 cells and culture-activated primary human HSCs. In vivo study results indicated that neratinib inhibited the inflammatory response, HSC differentiation, and collagen accumulation induced by CCl_4_. Moreover, the anti-fibrotic effects of neratinib were not associated with the HER2 signaling pathways. Neratinib inhibited FGF2 expression in activated HSCs and serum FGF2 level in the model, suggesting that neratinib possessed therapeutic potency against liver fibrosis and the potential for application against other fibrotic diseases.

## Introduction

The pathogenesis of liver fibrosis has been extensively studied for decades to identify effective therapies for this disease. The characteristic of liver fibrosis is the net accumulation of extracellular matrix (ECM) proteins caused by hepatic injuries, such as viral infection, alcohol, Nonalcoholic Steatohepatitis (NASH), autoimmune disorders, cholestatic disorders, and metabolic diseases^1^. During the early stage of liver disease, the inflammatory response and hepatic injury are initiated, and persistent inflammation in the liver leads to pathological accumulation of ECM proteins, such as collagens, laminin, and fibronectin^2^, which are responsible for cell migration and proliferation. Accumulation of excessive ECM proteins results from the abnormal regulation of ECM-degrading matrix metalloproteinases (MMPs) and their specific inhibitors (TIMPs)^3^.

Hepatic stellate cells (HSCs) are the primary effector cells of liver fibrosis with an ability to be transdifferentiated into myofibroblasts-like (MFB) cell types by hepatic injury^4,5^. Activated HSC (aHSC) consists of 90% ECM-producing MFBs resulting in the synthesis and secretion of matrix proteins that cause parenchymal and cholestatic liver damage^6–9^. In the healthy human liver, HSC represents 5–8% of total liver cells and has the function of vitamin A storage within cytoplasmic lipid droplets^10^. HSC is non-proliferative under normal conditions, but after chronic liver injury, it is transdifferentiated into the MFB phenotype to activate the immune response and angiogenesis^4,11^. aHSCs are characterized by cell proliferation, fibrogenesis, matrix degradation, contractility, and pro-inflammatory activity. Upon liver damage, aHSCs secrete platelet-derived growth factor (PDGF) and transforming growth factor (TGF-β), resulting in the activation of other hepatic cells, such as sinusoidal endothelial cells, Kupffer cells, and hepatocytes^2^. Type I, III collagen, α-smooth muscle actin (α-SMA), TGF-β, and tissue inhibitors of metalloproteinase (TIMP)-1 are considered well-characterized markers of HSC activation^5,12,13^.

This study aims to find druggable compounds targeting aHSCs to attenuate liver fibrosis without causing normal cell toxicity. Drug repurposing provides evidence for FDA-approved drugs that may also be effective in treating other diseases. To this end, neratinib was identified as a potential active candidate by cell-based screening using a kinase inhibitor library in HSCs. Neratinib (NERLYNX; Puma Biotechnology, Inc.) is an irreversible tyrosine kinase inhibitor that targets the human epidermal growth factor receptor (EGFR), HER2, and HER4. Currently, it has been approved by the FDA for early HER2-positive breast cancer in extended adjuvant treatment, and it is in ongoing clinical trials for lung, colorectal, and bladder cancers. Although diarrhea is considered an adverse effect, it is predictable and manageable with antidiarrheal medicine^14^. We determined whether neratinib mitigates liver fibrosis in a mouse model of carbon tetrachloride (CCl_4_)-induced fibrosis. To verify this hypothesis, we examined whether neratinib inhibits the profibrogenic activity of LX-2 cells and the activation of primary human stellate cells (hpHSCs). We also discussed the growth factor signaling to elucidate the underlying molecular mechanisms involved in HSC activation.

## Results

### Inhibitory effects of neratinib on HSC activation

**Figure 1:**
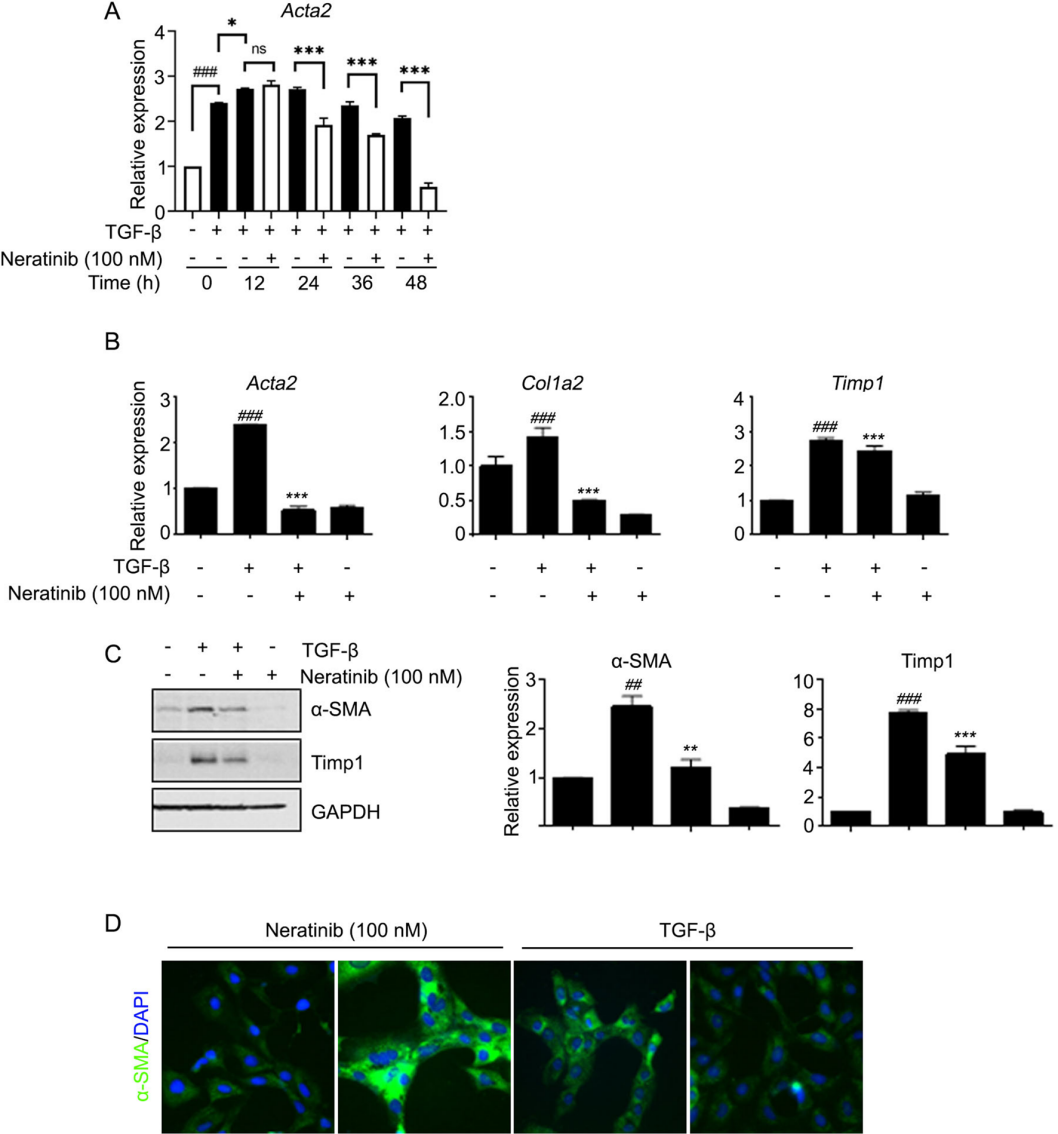
Neratinib decreased the pro-fibrogenic activity of LX-2 human hepatic stellate cells (HSCs). (**A**) TGF-β (5 ng/mL) was added to LX-2 cells for 48 h before neratinib treatment. The effect of 100 nM neratinib over various periods (0, 12, 24, 36, and 48 h) on LX-2 cells was assessed by qPCR analysis. TGF-β itself was used as a positive control. (**B**) qPCR and (**C**) western blotting were conducted after neratinib treatment (100 nM) for 48 h in the absence or presence of TGF-β (5 ng/mL). GAPDH was used as the loading control. (**D**) Immunofluorescence micrographs of LX-2 cells stained for α-SMA (green), nuclei (DAPI, blue), and merged (100× magnification; scale bar: 100 μm). The data are expressed as the mean ± S.E.M. ^*##*^*P* < 0.01, ^*###*^*P* < 0.001 versus the non-treated group. **P < 0.01, ***P < 0.001 versus the TGF-β-treated group.

**Figure 2:**
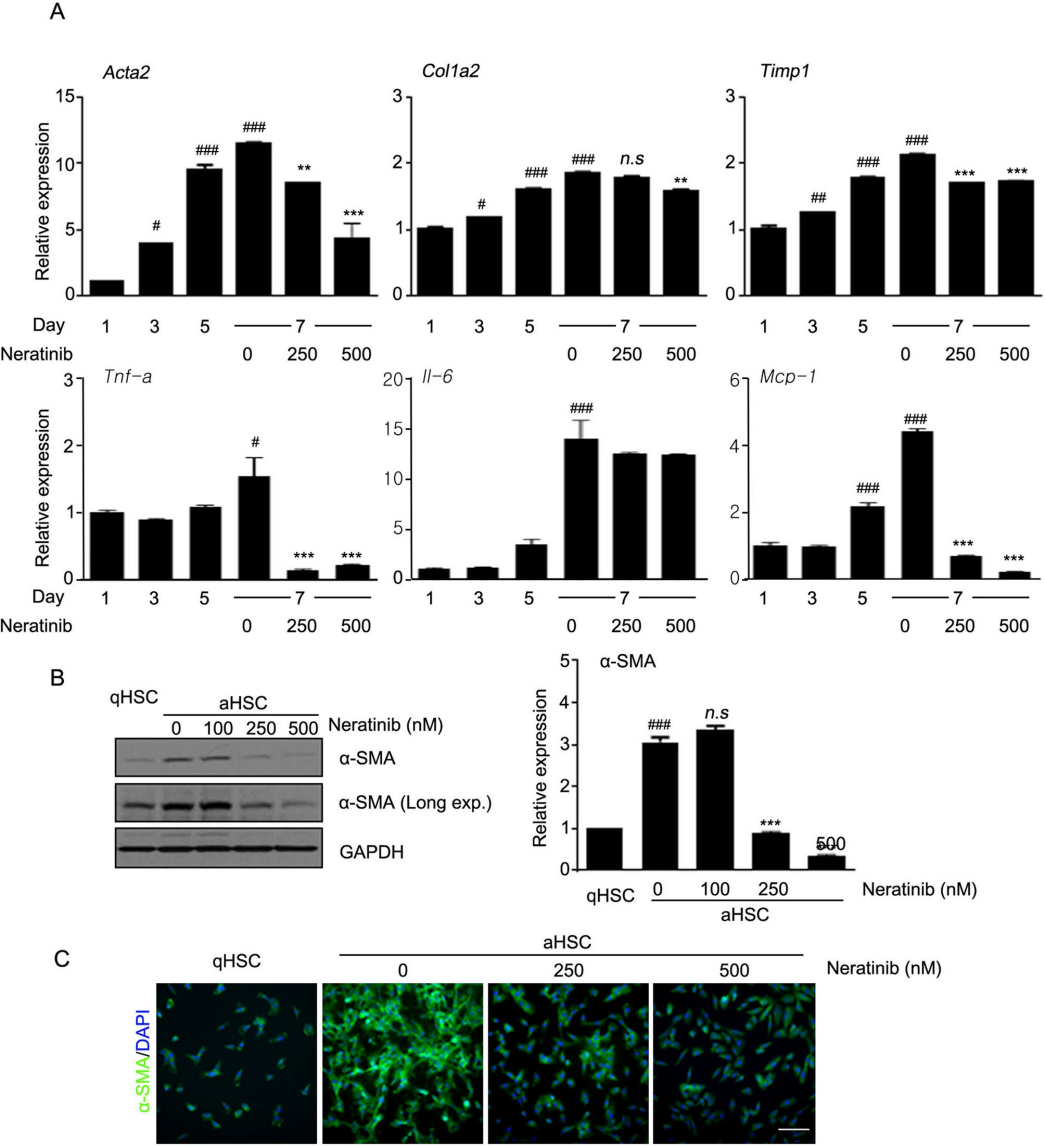
Neratinib decreased the activation of primary human HSCs (hpHSCs). hpHSCs were culture activated, and the effect of neratinib on aHSCs was analyzed by (**A**) qPCR and (**B**) western blotting after 48 h of neratinib treatment on day 7. GAPDH was used as the loading control. (**C**) Immunofluorescence micrographs of hpHSCs stained for α-SMA (green), nuclei (DAPI, blue), and merged (40× magnification; scale bar: 200 μm). qHSCs and aHSCs were harvested on day 3 and day 7, respectively. The data are expressed as the mean ± SEM. ^*###*^*P* < 0.001 versus the non-treated group. *P < 0.05, **P < 0.01, ***P < 0.001 versus the activated group**.**

The inhibitory effect of neratinib was examined in LX-2 cells, an immortalized aHSC line. It is well known that TGF-β contributes to HSC activation through the upregulation of excessive ECM deposition^15^. After incubation with TGF-β (5 ng/mL) for 48 h, the cells were treated with 100 nM neratinib (a non-cytotoxic concentration) at various times up to 48 h (Supplementary Fig. 1A, Fig. 1A). The mRNA level of *Acta2*, a representative marker of aHSCs, was significantly reduced by neratinib treatment in a time-dependent manner compared to TGF-β only treated positive control (Fig. 1A). mRNA levels of *Acta2*, *Col1a2*, and *TIMP-1* and protein expression of α-SMA and TIMP-1 were decreased when TGF-β was added in LX-2 cells to increase the pro-fibrogenic efficacy followed by neratinib for 48 h (Fig. 1B,C). Immunocytochemistry (ICC) results also indicated that neratinib significantly inhibits the effects of TGF-β on LX-2 cells (Fig. 1D). Next, the inhibitory effects of neratinib on HSC activation was confirmed in primary human HSCs (hpHSCs). We found that the neratinib incubation in hpHSCs showed less cytotoxicity than in LX-2 cells (Supplementary Fig. 1B). Moreover, neratinib showed negligible hepatocyte toxicity when the isolated mouse primary cells were treated with varying concentrations of neratinib (Supplementary Fig. 1C). Next, hpHSCs were culture-activated for 3 (quiescent), 5, or 7 days (fully activated) to decide the levels of fibrogenic and proinflammatory mRNA during the activation. hpHSCs were treated with a non-cytotoxic concentration of neratinib (250 or 500 nM) to determine anti-fibrotic effects by qPCR for mRNA levels of *Acta2* (α-smooth muscle actin), *Col1a2* (collagen I), *TIMP-1* (tissue inhibitor of metalloproteinase-1), *TNF-a* (tumor necrosis factor α), *Il-6* (interleukin 6), *and Mcp-1* (monocyte chemoattractant protein 1) (Fig. 2A). Interestingly, Neratinib suppressed the mRNA level of Mcp-1, which is known to be expressed in aHSC. Western blotting analysis showed that the protein expression of α-SMA also decreased by neratinib treatment from 250 nM (Fig. 2B). ICC showed a significant increase in α-SMA expression in culture-activated hpHSCs. In contrast, neratinib treatment suppressed the increase in a dose-dependent manner, consistent with the results of gene expression analysis (Fig. 2C). These results showed that neratinib is a potential candidate for liver fibrosis treatment targeting HSCs.

### Anti-fibrotic effects of neratinib against CCl_4_-induced early-stage liver fibrosis in mice

**Figure 3:**
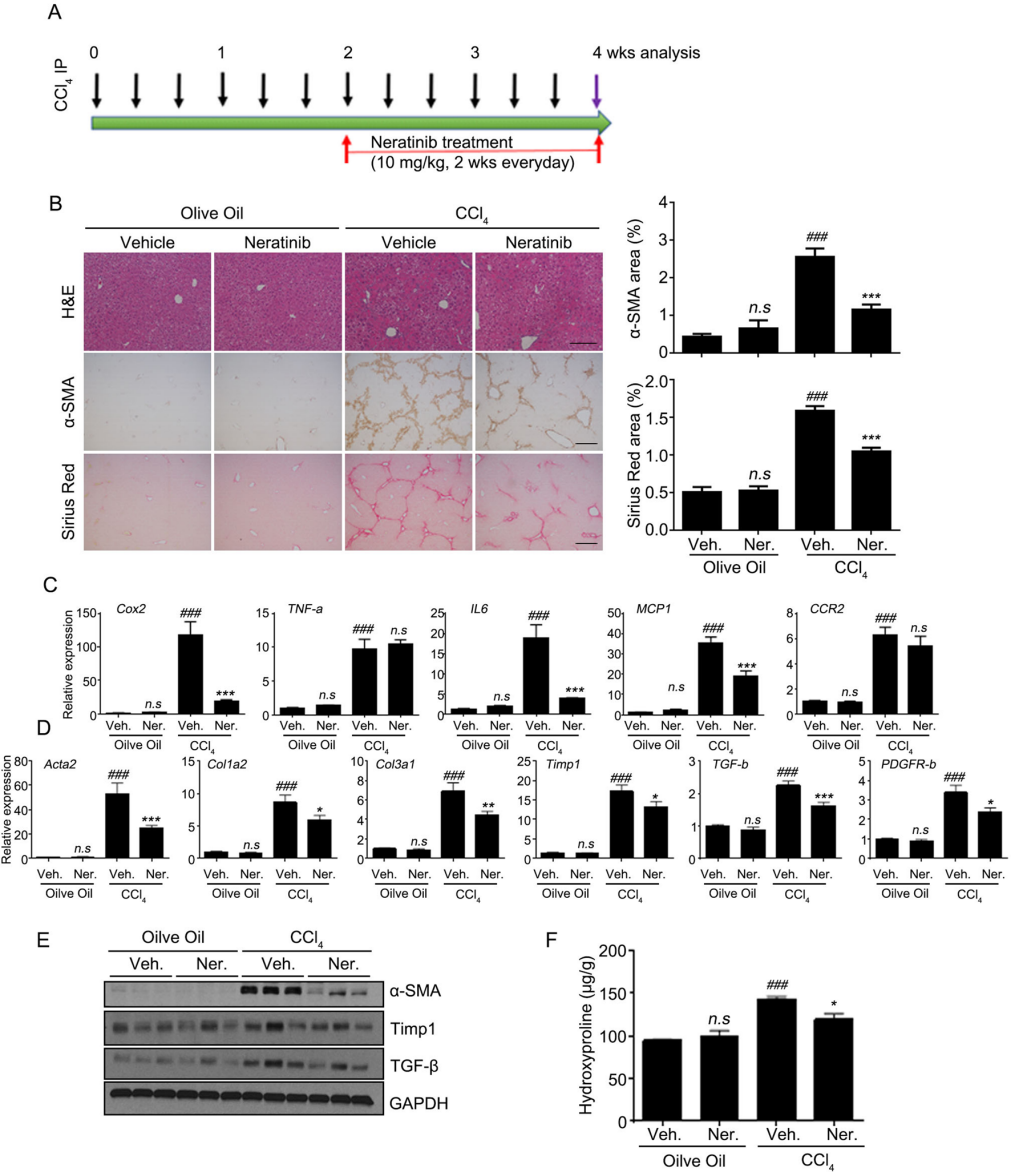
Neratinib ameliorated the early stage of fibrosis in a CCl_4_-induced mouse model. (**A**) To examine the effects of neratinib in the early stage of liver fibrosis, neratinib (10 mg/kg) was orally administered every day for 2 weeks in the control (vehicle-treated) and liver fibrosis (induced by 2 weeks CCl_4_ treatment [20% CCl_4_, 2 mL/kg, three times per week]) groups. (**B**) Representative photomicrographs of liver tissue sections stained with H&E, α-SMA, and Sirius Red. (H&E: 100× magnification; scale bar: 100 μm. α-SMA, Sirius red: 40× magnification; scale bar: 200 μm). The percentages of α-SMA- and Sirius red-positive cells were measured in six random fields. (**C**) Gene qPCR analysis of the liver tissues in each group (n = 5). mRNA levels of inflammatory markers (*Cox-2*,* TNF-α*, *IL-6*, *MCP-1*, and *CCR-2*) and (**D**) fibrotic markers (*Acta2*, *Col1a2*, *Col3a1*, *TIMP-1*, *TGF-b*, and *PDGFR-b*). (**E**) Western blotting results of α-SMA, TGF-β, TIMP-1, and PDGFR-β in the livers of mice from each group. GAPDH was used as the loading control. (**F**) Liver hydroxyproline content was assessed to determine the total collagen content in the liver of mice from each group. Data were compared to the vehicle-treated liver fibrosis group. Abbreviations: Veh, vehicle; Ner, neratinib.

With the confirmation of an inhibitory effect of neratinib on HSC activation, the anti-fibrotic effect in vivo was investigated using a mouse model of liver fibrosis induced by repeated injections of CCl_4_. After repeated injection of CCl_4_ for 4 and 10 weeks, which corresponded to the early and advanced stages of liver fibrosis, respectively, mice were randomized into four groups and orally administered control vehicle or neratinib while continuing to receive CCl_4_ injections. In the early stages of fibrosis, neratinib (10 mg/kg) was administered orally (P.O.) for 2 weeks daily after induction of fibrosis with CCl_4,_ as shown in the treatment schedule in Fig. 3A. Microscopy analysis of hematoxylin and eosin (H&E) staining showed that CCl_4_ promotes exacerbation of the damaged area with signs of hepatic fibrosis. Next, immunohistochemistry (IHC) analysis was performed to investigate the pathophysiological efficacy of neratinib in the liver tissues. IHC and quantified digital analyses showed that the positive area of α-SMA staining and collagen deposition (Sirius Red) in the liver of neratinib-treated mice were significantly reduced compared to that of the vehicle-treated group (Fig. 3B). We found that the expression of the target mRNA and proteins were much lower in the liver treated with neratinib than in the vehicle-treated group when the anti-inflammatory/anti-fibrotic effect was determined by qPCR and western blotting. It is well known that chronic inflammation drives hepatic fibrosis^16^. The disease group showed high levels of pro-inflammatory cytokines, including *Cox-2* (cyclooxygenase 2), *TNF-a*, *IL-6*, *MCP-1*, and *CCR-2* (C–C chemokine receptor 2) mRNA. However, neratinib treatment strongly suppressed the levels of mRNA, except for *TNF-a* and *CCR-2* (Fig. 3C). More importantly, neratinib treatment reversed the increased gene levels of fibrotic markers, namely *Acta2*, *Col1a2*, *Col3a1* (collagen III), *TIMP-1*, *TGF-b*, and *PDGFR-b* (platelet-derived growth factor β) (Fig. 3D). Western blotting analysis showed that the expression of α-SMA, TIMP-1, and TGF-β decreased in the neratinib-treated group compared to that in the vehicle-treated group, supporting the anti-fibrotic effects of neratinib in the disease model (Fig. 3E). Moreover, the hydroxyproline level for collagen contents was much lower in the neratinib-treated group than in the vehicle-treated group (Fig. 3F). These findings suggest that neratinib prevents the early stage of liver fibrosis with an anti-fibrotic/anti-inflammatory effect.

### Neratinib ameliorates the advanced stage of liver fibrosis induced by CCl_4_

**Figure 4:**
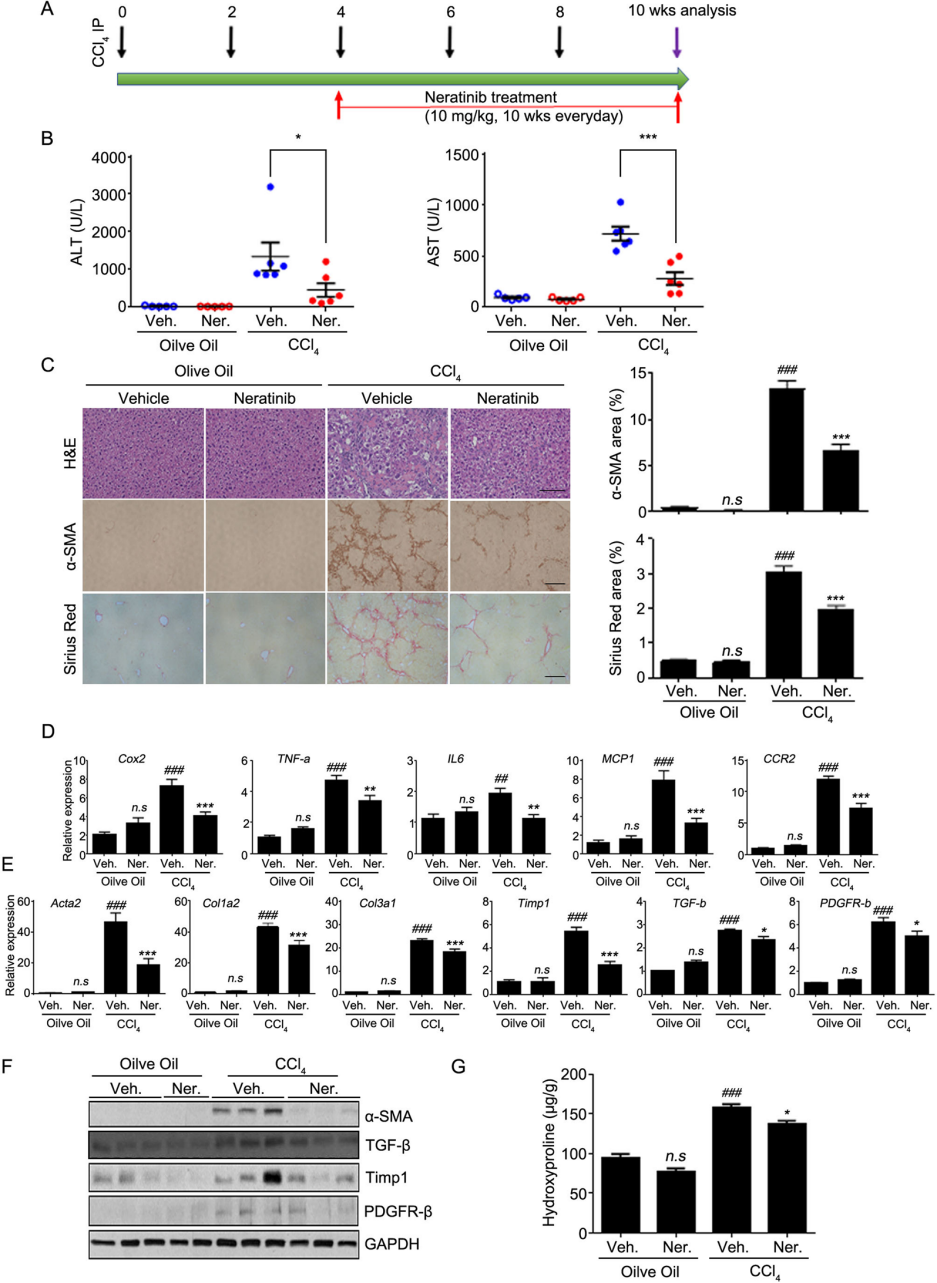
Neratinib treatment ameliorated the advanced stage of fibrosis in a CCl_4_-induced mouse model. (**A**) To examine the effects of neratinib on the advanced stage of liver fibrosis, neratinib (10 mg/kg) was orally administered daily for 6 weeks in the control (vehicle-treated) and liver fibrosis (induced by 4 weeks CCl_4_ treatment [20% CCl_4_, 2 mL/kg, three times per week]) groups. (**B**) Serum levels of alanine transaminase (ALT) and aspartate transaminase (AST) were analyzed. (**C**) Representative photomicrographs of liver tissue sections stained with H&E, α-SMA, and Sirius Red (H&E: 100× magnification; scale bar: 100 μm. α-SMA, Sirius Red: 40 × magnification; scale bar: 200 μm). The percentages of α-SMA- and Sirius Red-positive cells was measured in six random fields. mRNA levels of (**D**) inflammatory markers (*Cox-2*, *TNF-α*, *IL-6*, *MCP-1*, and *CCR-2*) and (**E**) fibrotic markers (*Acta2*, *Col1a2*, *Col3a1*, *TIMP-1*, *TGF-b*, and *PDGFR-b*) were measured by qPCR analysis of liver tissues in the vehicle-treated healthy group, neratinib-treated healthy group, vehicle-treated liver fibrosis group, and neratinib-treated liver fibrosis group (n = 5–6). (**F**) Western blotting results for α-SMA, TGF-β, TIMP-1, PDGFR-β, and GAPDH in the livers of mice from each group. GAPDH was used as the loading control. (**G**) Liver hydroxyproline content was assessed to determine the total collagen content in the liver of mice from each group. Data are expressed as the mean ± S.E.M. ^*##*^*P* < 0.01, ^*###*^*P* < 0.001 versus the vehicle-treated healthy group. *P < 0.05, **P < 0.01, ***P < 0.001 versus the vehicle-treated liver fibrosis group. Abbreviations: Veh, vehicle; Ner, neratinib.

After demonstrating clear anti-fibrotic activity of neratinib in the early-stage liver fibrosis, we conducted further experiments in the advanced stage of liver fibrosis. In the advanced stage of fibrosis after 4 weeks of CCl_4_ administration, as shown in Fig. 4A, mice were treated with neratinib (10 mg/kg) P.O. for 6 weeks. Blood was collected for analysis of alanine transaminase (ALT) and aspartate aminotransferase (AST) levels at the end of the study. Both serum ALT and AST levels markedly increased after CCl_4_ administration, but significantly decreased in the sera of neratinib-treated mice compared to that in the vehicle-treated mice (Fig. 4B). Analysis of H&E staining showed fibrosis, necrotic lesions, and inflamed area in the hepatic lobule portal area of the advanced stage fibrosis model (Fig. 4C). IHC and quantified digital image analyses showed an apparent reduction of positive area for fibrotic markers (staining for α-SMA antibodies and collagen deposition) in liver specimens from neratinib-treated mice compared with those in vehicle-treated mice (Fig. 4C). Next, the mRNA levels and protein expression of pro-inflammatory cytokines and fibrotic markers in liver tissues were analyzed. Compared to the early fibrosis model, the levels of mRNA targeting inflammation were not increased much in the advanced fibrosis group, but still, neratinib treatment strongly suppressed the levels of mRNA of inflammatory cytokines induced by CCl_4_ (Fig. 4D). qPCR analysis showed a decrease in highly upregulated gene levels associated with HSC activation, namely *Acta2*, *Col1a2*, *Col3a1*, *TIMP-1*, *TGF-b*, and *PDGFR-b*, in the neratinib-treated group compared to that in the vehicle-treated fibrosis group (Fig. 4E). Western blotting analyses confirmed a significant reduction in the protein expression of α-SMA, TGF-β, Timp-1, and PDGFR-β in the neratinib-treated group compared to those in the vehicle-treated group (Fig. 4F). Importantly, the hydroxyproline assay showed a much lower level of collagen contents in the neratinib-treated group than in the vehicle-treated liver fibrosis group (Fig. 4G). These findings indicate that neratinib treatment functionally relieves CCl_4_-induced advanced liver fibrosis to early-stage fibrosis.

### Neratinib inhibits the MEK-ERK-CREB signaling pathway in activated human primary HSCs

**Figure 5:**
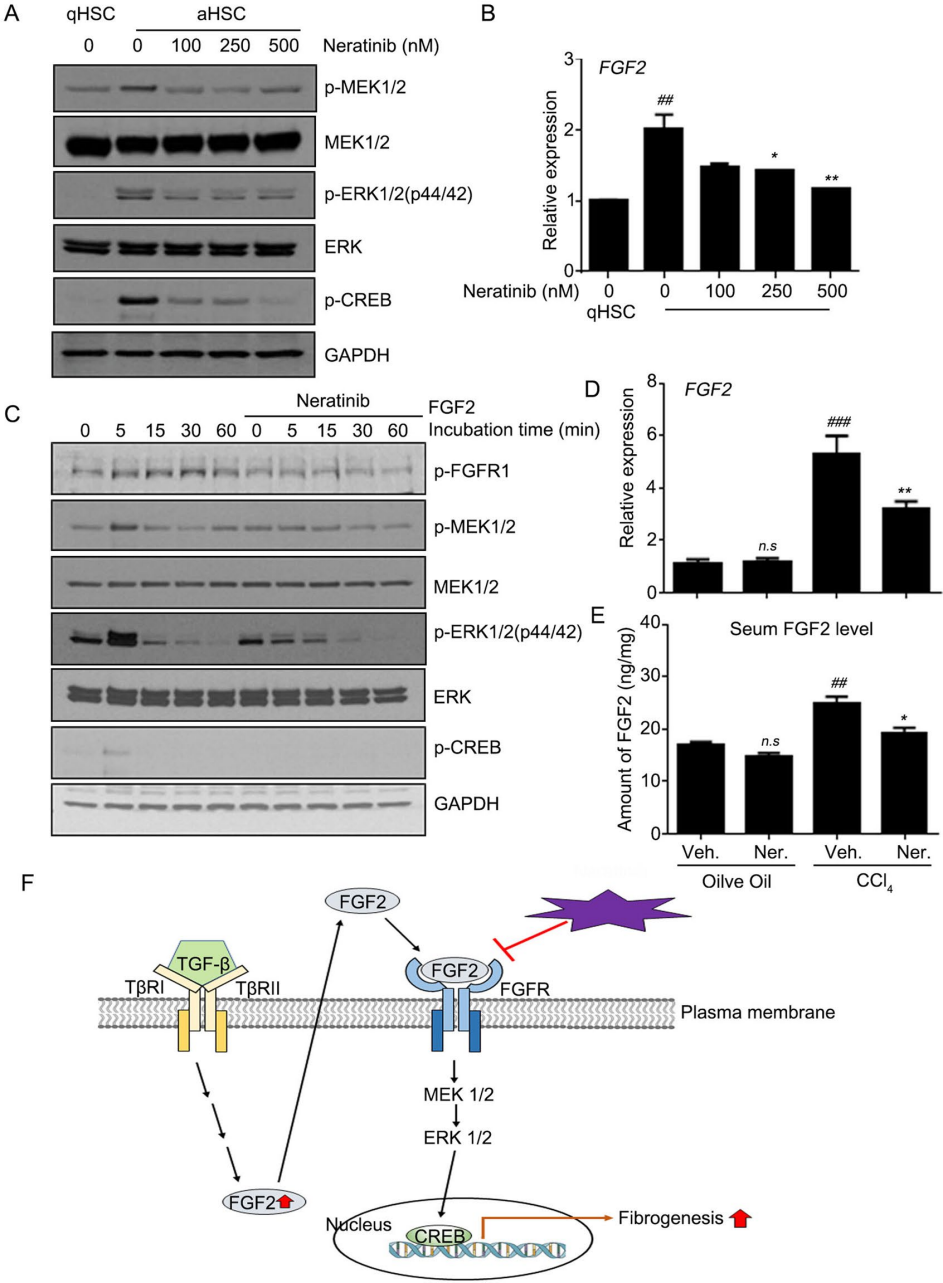
Neratinib inhibited fibrogenesis by interrupting the FGF2 signaling pathway. (**A**) Neratinib inhibited the induction of the MAPK/ERK signaling pathway in activated hpHSCs. (**B**) The effect of neratinib on *FGF2* mRNA level in activated hpHSCs was measured. (**C**) hpHSCs were serum-starved overnight before exposure to neratinib (250 nM) in the absence or presence of FGF2 (10 ng/mL). Western blotting for p-FGFR1, p-MEK1/2, MEK1/2, p-ERK, ERK, and p-CREB was performed. GAPDH was used as the loading control. (**D**) The effects of neratinib on *FGF2* mRNA expression and (**E**) serum FGF2 levels were analyzed in the early fibrosis mouse model. ^*##*^*P* < 0.01, ^*###*^*P* < 0.001 versus the non-treated group. *P < 0.05, **P < 0.01 versus the activated group. (**F**) Scheme of the proposed working model of neratinib in liver fibrosis. Data are expressed as the mean ± SEM. Abbreviations: qHSCs, quiescent HSCs; aHSCs, activated HSCs; TβRI, TGF-β receptor 1; TβRII, TGF-β receptor 2; Veh, vehicle; Ner, neratinib.

It is well known that TGF-β activates hepatic cells, promoting the secretion of ECM components and resulting in fibrotic livers. TGF-β binding to the cell-surface receptor TβRI initiates the intracellular signaling cascade^15,17^. Following TβRI phosphorylation, the intracellular Smad signal transducer proteins, including Smad2/3, are phosphorylated and translocated into the nucleus to regulate the transcription of the target gene. Increasing evidence implicates noncanonical TGF-β signaling pathways in liver fibrosis, although the Smad-dependent TGF-β signaling pathway is widely recognized in fibrotic conditions^18,19^. We investigated whether neratinib participates in the Smad-dependent pathway in regulating HSC activation while also focusing on Smad-independent signaling, mitogen-activated protein kinase (MAPK) pathways via the ERK/CREB pathway. As expected, Smad2 and Smad3 were highly phosphorylated in LX-2 cells exposed to TGF-β. However, pre-treatment with neratinib did not affect this phenomenon (Supplementary Fig. 2A). Subcellular fractionation was employed to confirm the role of neratinib on the translocation of Smads in activated HSCs. Nuclear and cytoplasmic fraction was prepared after stimulation with TGF-β in LX-2 cells in the absence or presence of neratinib. Consistent with the phosphorylation results, neratinib did not prevent the nuclear translocation of Smad (Supplementary Fig. 2B,C). Notably, we found that activated hpHSCs did not express HER-2 protein when BT474 positive cells were used as a positive control (Supplementary Fig. 2D). Next, we observed the Smad-independent ERK/CREB pathway in the activated hpHSCs (Fig. 5A). We speculated that neratinib might prevent fibrogenesis through the ERK/CREB pathway. To verify this hypothesis, cultured activated hpHSCs were treated with different doses of neratinib for 48 h and then subjected to western blotting using MEK/ERK/CREB antibodies. We showed that neratinib inhibited the phosphorylation of MEK, ERK, and CREB in activated hpHSCs (Fig. 5A). Furthermore, increasing evidence shows that fibroblast growth factors (FGFs) are the key regulator of HSCs that act in an autocrine and paracrine manner. Besides, mRNA levels for the FGF family in activated hpHSC were investigated. Among the FGF family genes, FGF1 and 2 were the most potential candidates, as their gene levels were significantly reduced by neratinib treatment (Fig. 5B, Supplementary Fig. 3). Notably, it has been reported that fibrotic liver tissue highly expresses the FGF2 protein, but not in a healthy liver. Other studies have shown that FGF2‐deficient mice exhibit reduced liver fibrosis after CCl_4_ administration, suggesting that FGF2 is a potential candidate profibrogenic factor. When hpHSCs were incubated with FGF2 protein in the presence or absence of neratinib, FGF2 increased the levels of the MEK1/2, ERK, and CREB phosphorylation in addition to FGF2 phosphorylation within 5 min incubation. As we expected, neratinib inhibited the phosphorylation (Fig. 5C). Finally, qPCR analysis and ELISA showed that increased expression levels of FGF2 in CCl_4_ mice were significantly decreased in the neratinib-treated group (Fig. 5D,E). These findings demonstrate that neratinib targets downstream of TGF-β by inhibiting the FGF2-mediated MAPK signaling (Fig. 5F). A kinase inhibitor, neratinib, exerts a protective effect against liver fibrosis by selectively targeting HSC, the originators of liver fibrosis.

## Discussion

Neratinib was approved by the FDA for the treatment of HER2-positive breast cancer. Clinical studies have reported that neratinib affects not only breast cancer but also non-small cell lung cancer, colorectal cancer, and biliary cancer. In this study, we presented the first evaluation of the anti-fibrotic effects of neratinib on the liver by examining its efficacy in inhibiting HSC activation. Neratinib showed potent inhibitory effects on HSC activation in vitro*.* To evaluate the anti-fibrotic effects, neratinib was orally administered to mice with CCl_4_-induced liver fibrosis, and the results showed strong anti-fibrotic effects in the early and advanced stages of liver fibrosis. The signaling pathways associated with HSC activation were explored to elucidate the mechanism of action.

The accumulation of ECM proteins is a result of the wound healing process that occurs after chronic inflammation induced by liver injury^2,4^. In the past several decades, there has been considerable progress in managing liver fibrosis, with various factors, including alcohol consumption, hepatitis B virus (HBV), hepatitis C virus (HCV), and metabolic disorders, identified as significant causes of liver fibrosis^3^. Prolonged liver fibrosis leads to liver cirrhosis, liver failure, hepatocellular carcinoma, and death^20^. Recently, several drugs, including antiviral drugs, have been approved by the FDA for liver fibrosis. However, these drugs are only useful in some circumstances, and there is currently no successful liver-specific fibrosis therapies^21^. Also, there are no FDA-approved small molecules targeted anti-fibrotic therapy except Mulpleta (lusutrombopag), which stimulates the production of platelets. Therefore, the development of an anti-fibrotic medication is indispensable for the regression of advanced fibrosis through inhibition of the source of liver fibrosis.

Our results show that neratinib ameliorates CCl_4_-induced liver fibrosis in the early and advanced stages by inhibiting HSC activation. aHSCs are the primary effector cells in liver fibrogenesis, contributing approximately 90% of ECM-producing myofibroblasts^2,7,22^. Inhibition of HSC activation and removal of aHSCs have been studied as primary approaches for liver fibrosis^23–25^. It would be a logical strategy to develop the small molecule targeting aHSC in chronic liver diseases to improve the treatment efficacy. Neratinib was selected as a drug with potent inhibitory effects against fibrogenesis in hpHSCs and LX-2 cells after kinase inhibitor library screening.

Neratinib is an irreversible, pan-HER tyrosine kinase inhibitor recently approved by the FDA as an extended adjuvant treatment for patients with HER2^+^ early-stage breast cancer that does not cause severe adverse effects, except diarrhea^26^. In addition, neratinib shows anticancer effects in non-small cell lung cancer, colorectal cancer, and glioblastoma through the EGFR/HER-2 pathway^26–28^. Several studies reported that EGFR/HER-2 inhibitors attenuated liver fibrosis through inhibition of HSCs^29,30^. However, we found that activated hpHSCs do not express HER-2 protein (Supplementary Fig. 2D). Instead, we found that neratinib blocked MEK-ERK-CREB signaling in activated HSCs. Moreover, FGF2 is a member of the FGF1 subfamily, and its role in fibrogenesis is unclear. A high level of FGF2 was observed in the serum and liver tissue of patients with chronic liver disease, but not in the normal liver^31,32^. Lin et al*.* and Yu et al*.* suggested that FGF2 induced the expression of collagen and α-SMA, and depletion of FGF2 decreased CCl_4_-induced hepatic fibrosis^33,34^. However, Sato-Matsubara et al*.* insisted that FGF2 ameliorated BDL mice and inhibited HSC activation. Koo et al*.* showed that FGF2 inhibited fibroblast collagen production and myofibroblast differentiation, which suggested that FGF2 is a potential candidate for the pro-fibrotic factor^35,36^. In our study, the levels of *FGF2* mRNA and serum FGF2 were increased in the liver of CCl_4_-treated mice, and aHSCs showed increased the levels of *FGF2* mRNA.

In vivo results strongly indicated that neratinib had inhibitory ability against hepatic fibrogenesis. CCl_4_ is a widely used toxic chemical in liver fibrosis studies, and it acts by increasing the levels of free radicals^37,38^. The first symptom of histological fibrosis is usually observed after 2–3 weeks of CCl_4_ injections and fibrosis can be detected at the molecular level between 4 and 6 weeks after injection, depending on the dosage, in C57BL/6 mice^39^. Our results showed that fibrogenic markers (α-SMA and collagen) significantly increased at the early stage of liver fibrosis, and the expression of these markers was higher at the advanced stage. In contrast, the expression of inflammatory cytokines was much lower at the advanced stage than at the early stage. Our results show that neratinib treatment significantly ameliorates both stages of liver fibrosis mice, as confirmed by the reductions in α-SMA expression, collagen deposition, and other fibrogenic markers (TIMP-1, PDGFR-β, and TGF-β) in liver tissues. The levels of mouse IL-6 partially blocked when we conducted an additional study using isolated primary mouse Kupffer cells after co-treatment with Neratinib/LPS for 4 h, indicating that neratinib may also directly target immune cells (Supplementary Fig. 4). A recent study showed that neratinib suppressed the mRNA level of IL-6, TNF-a, and IL-1b in mouse macrophage 264.7 with LPS activation^40^.

Our results revealed that neratinib ameliorates liver fibrogenesis through the suppression of aHSCs. Also, this study suggests that neratinib targets the inhibition of FGF2 as a novel candidate to alleviate aHSC, a potential target for liver fibrosis.

## Methods

### CCl_4_-induced liver fibrosis mouse model

The animal experimental procedures were conducted following the Guidelines of Animal Use and Care of the National Institute of Health, and they were approved by the Johns Hopkins Animal Care and Use Committee. All efforts were made to minimise the suffering of animals. Male C57/BL6 mouse (age, 5–6 weeks; body weight, 20–30 g) were purchased from Charles River (Germantown, MD). The mice were randomly divided into four groups: (1) vehicle-treated healthy group, in which mice received olive oil and vehicle treatment (0.5% methylcellulose-0.2% Tween 80), (2) neratinib-treated healthy group, (3) vehicle-treated liver fibrosis group, and (4) neratinib-treated liver fibrosis group.

To evaluate short-term treatment of liver fibrosis, the mice were treated with 2 mL/kg CCl_4_ (Sigma-Aldrich, St. Louise, MO, USA; 20% CCl_4_ in olive oil) three times per week i.p. or with olive oil (as a control) for a total of 6 weeks. On day 15, mice were treated with 10 mg/kg neratinib or vehicle p.o. daily for 14 days. Mice were anesthetized on day 28, and blood and liver tissue samples were collected for analysis. In the long-term treatment of liver fibrosis, the mice were treated with 2 mL/kg CCl_4_ three times per week for 4 weeks before neratinib treatment. On day 29, the mice were treated with 10 mg/kg of neratinib or vehicle p.o. daily for 42 days. Mice were anesthetized on day 70, and blood and liver tissue samples were collected for analysis.

### Mouse blood analysis

Blood samples were collected by cardiac puncture, stored at 20–25 °C for 2 h, and then centrifuged at 10,000 rpm for 10 min. The levels of the liver enzymes AST and ALT in serum were analyzed as a measure of liver function. Serum FGF level was analyzed by using an FGF2 Quantikine ELISA kit (MFB00; R&D Systems, Minneapolis, MN, USA) following the manufacturer's protocol.

### Liver histology, immunohistochemistry, and immunofluorescence analyses

Liver tissues were fixed in 10% buffered formalin, embedded in paraffin, and cut into 4 µm-thick sections. The sections were then stained with H&E and Sirius Red by standard protocols. The amount of collagen was quantified from Sirius Red staining by using an image processing software (ImageJ; NIH). For IHC staining, anti-α-SMA (A2547; Sigma-Aldrich) antibodies were used to detect activated HSCs. IHC was performed following a standard protocol, and the stained tissues were quantified from 20 randomly selected images from each section.

### Hepatic hydroxyproline measurement

Collagen level in the liver was measured by using a hydroxyproline assay kit (MAK008; Sigma-Aldrich) following the manufacturer's protocol. Briefly, approximately 10 mg of liver tissues were homogenized, hydrolyzed at 120 °C for 3 h in 12 N HCl, incubated with 100 μL Chloramine T/Oxidation buffer mixture for 5 min, and then incubated with DMAB reagent for 90 min at 60 °C. The absorbance at 560 nm was measured by using a microplate reader (Bio-Tek Instruments, Inc., Winooski, VT).

### Cell culture

As described our previous report^41^, LX-2 cells were maintained in Dulbecco's Modified Eagle's Medium (Sigma-Aldrich) supplemented with 10% fetal bovine serum (FBS) and 1% penicillin/streptomycin solution. hpHSC was purchased from ScienCell Research Laboratories (Carlsbad, CA), and cultured in stellate cell medium supplemented with 2% FBS, 1% stellate cell growth supplement, and 1% penicillin/streptomycin solution in poly-l-Lysine-coated plates. The hpHSCs were seeded in a six-well culture plate for 1, 3, 5, and 7 days and harvested for analysis. The expression of the activation marker α-SMA in cultured stellate cells was determined by western blotting and qPCR analyses. Cells were cultured at 37 °C in an atmosphere of 5% CO_2_/95% air under saturating humidity.

### Hepatocyte isolation

Seven weeks of age mice were used for primary hepatocytes isolation as previously described^42^. Briefly, mouse liver was perfused via the portal vein with a 1X EGTA and then HBSS (Ca2+/Mg2+-free HBSS) containing 0.75 mg/mL of type I collagenase (Worthington Biochemical Corporation, NJ, USA). The liver was treated with a digestion buffer (0.08 mg/mL type I collagenase) and incubated for 15 min at 37 °C. The tissue went through 100 μm nylon cell strainer (Corning, USA) followed by three times of washing. 5 × 10^4^ cell/well of resuspended cells were plated at collagen-coated 96 well plates (Corning) in DMEM (Sigma-Aldrich, Logan, UT) with 10% of FBS and 1% antibiotics.

### Cytotoxicity assay

Cytotoxicity assay was performed using a CellTiter-Glo Luminescent Cell Viability Assay kit. After incubation with chemicals, cells were lyzed with lysis reagent, and an equal volume of CellTiter-Glo reagent was added to the cells. After incubation for 10 min at room temperature, luminescence was measured by using a luminometer (Bio-Tek Instruments, Inc.).

### Comparative qPCR analysis

As described our previous report^41^, total RNA from cells and liver tissues was extracted by TRIzol reagent (Life Technologies, Grand Island, NY) following the manufacturer's protocol. RNA concentration was measured by using a NanoDrop 2000 (Thermo Fisher Scientific, Waltham, MA). cDNA was synthesized from 1–2 µg of total RNA by using a High-Capacity cDNA Reverse Transcription System (Life Technologies). Comparative qPCR was performed in duplicate for each sample by using SYBR Green Master Mix (Life Technologies) and StepOnePlus Real-Time PCR System (Life Technologies). mRNA expression was quantified using the threshold cycle (Ct) method. Ct values for each gene of interest were normalized to that of GAPDH. The name and sequences of primers and primers for humans and mice are summarized in Supplementary Tables 1 and 2.

### Western blotting analysis

Anti-alpha SMA. (A2547; Sigma-Aldrich), anti-collagen (#90395; Abcam, Cambridge, MA), anti-TGF-β (#66043; Abcam), anti-PDGFR-β (sc-432; Santa Cruz Biotechnology, Santa Cruz, CA), anti-GAPDH (sc-1694; Santa Cruz Biotechnology), anti-p-MEK1/2 (#2338; Cell Signaling Technology, Danvers, MA), anti-MEK1/2 (#8727; Cell Signaling Technology), anti-p-ERK1/2(p44/42) (#4370; Cell Signaling Technology), anti-ERK (#4695; Cell Signaling Technology), and p-CREB (#9198; Cell Signaling Technology) were used for western blotting. Cell or tissues were lysed with RIPA buffer (G-Biosciences, St. Louis, MO, USA) with a protease inhibitor cocktail followed by western blotting as previously described^41^. The protein concentration of the lysates was quantified by using a BCA Protein Assay Kit (Pierce, Rockford, IL, USA) Denatured proteins were loaded into SDS–polyacrylamide gel, resolved by electrophoresis, and transferred to a nitrocellulose membrane, blocked by incubation with 3% bovine serum albumin (Sigma) for 1 h at room temperature, and the membrane was then incubated with each primary antibody overnight. Incubation with the appropriate secondary antibodies conjugated to HRP was performed for 1 h. Protein bands were developed onto an X-ray film (Fujifilm) after incubation with enhanced chemiluminescence.

### Enzyme-linked immunosorbent assays (ELISA) assay

TNF-α (SMTA00B) and IL-6 (SM6000B) were determined using Quantikine ELISA (R&D Systems). Neratinib was pretreated for 30 min followed by LPS (1 µg/mL, Sigma-Aldrich) for 4 h in mouse Kupffer. The collected supernatant was processed according to the manufacturer’s protocol.

### Statistical analysis

The GraphPad Prism 7 software (Graphpad Software, Inc., La Jolla, CA) and Excel 2016 (Microsoft, Redmond, WA) were used to analyze the data as previously described^41^. Differences between groups were assessed by one-way analysis of variance followed by Tukey’s *post-hoc* test or Student’s *t*-test, as appropriate. Error bars represent the standard deviation (SD) or standard error of the mean (SEM), as indicated. Statistical significance was accepted at *P* < 0.05.

## Supplementary information

Supplementary information

## Data Availability

Data available on request from the author.
