# Supplementary material for: Tyrosine kinase inhibitor neratinib attenuates liver fibrosis by targeting activated hepatic stellate cells
Source: Sci Rep. 2020 Sep 8;10:14756. doi: 10.1038/s41598-020-71688-2 (PMC7479613; doi:10.1038/s41598-020-71688-2)

**Tyrosine kinase inhibitor neratinib attenuates liver fibrosis by targeting activated hepatic stellate cells**

Yong Joo Park^1,2,4^, Hyoung-Tae An^1,2,3^, Jong-Sung Park^1,2^, Ogyi Park^1,2^, Alexander J. Duh^2^, Kwangmeyung Kim^3^, Kyu Hyuck Chung^4^, Kang Choon Lee^4^, Yumin Oh^1,2,*^, and Seulki Lee^1,2,*^

^1^ Russell H. Morgan Department of Radiology and Radiological Science, Johns Hopkins University School of Medicine, Baltimore 21205 MD, USA.

^2^ Center for Nanomedicine at the Wilmer Eye Institute, Johns Hopkins University School of Medicine, Baltimore 21205 MD, USA.

^3^ Center for Theragnosis, Korea Institute of Science and Technology, Seoul, Korea

^4^ School of Pharmacy, Sungkyunkwan University, Suwon 16419, Korea

**Corresponding authors**

Address correspondence to Yumin Oh (Yoh@neuralymed.com) or Seulki Lee (Slee343@jhmi.edu)


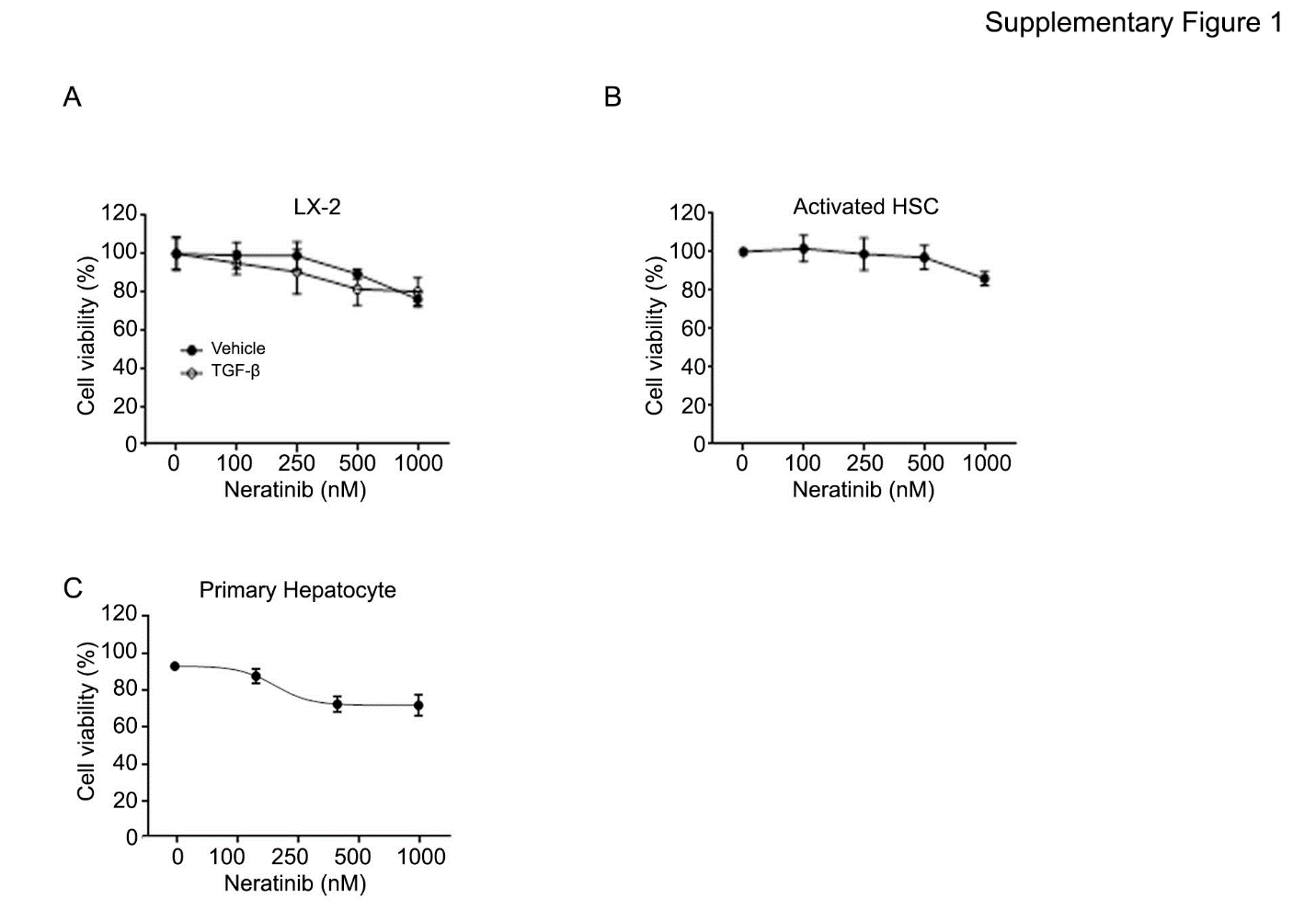


**Supplementary Figure 1.** Cytotoxicity of neratinib in LX-2 cells and activated hpHSC**.** (A) The percentage inhibition of LX-2 cell proliferation was determined after 48 h of treatment with DMSO (vehicle) or neratinib at different concentrations (100, 250, 500, and 1,000 nM) in the presence of TGF-β (5 ng/mL). (B) The percentage of viable cells in hpHSCs was determined after 48 h of treatment with neratinib at different concentrations (100, 250, 500, and 1,000 nM) on day 7. (C) Cell viability in primary mouse hepatocyte was determined after 24 h of treatment with neratinib at different concentrations (100, 250, 500 and 1,000 nM)


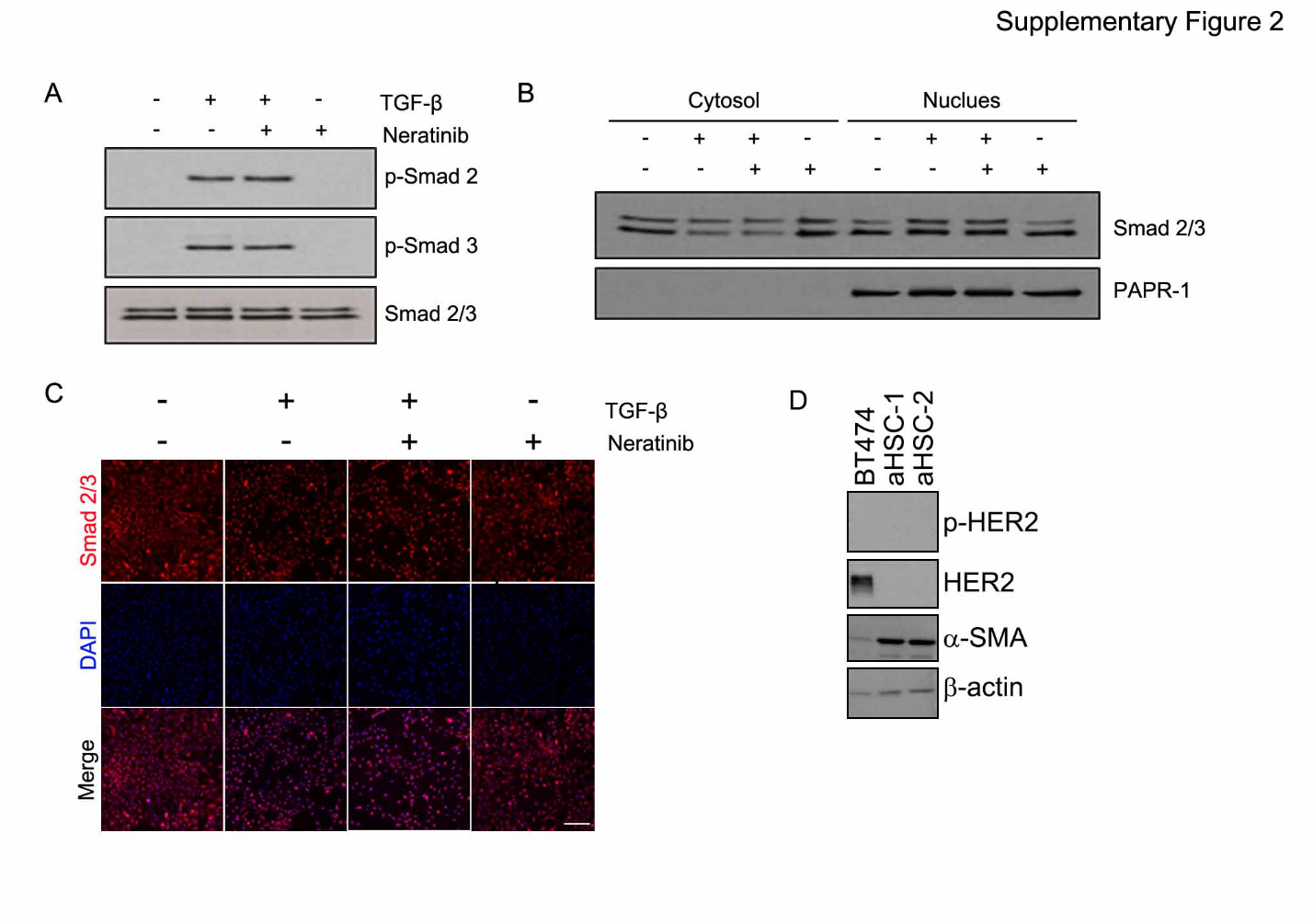


**Supplementary Figure 2.** Neratinib did not block the TGF-β/Smad signaling pathway. Neratinib (100 nM) was treated after TGF-β (5 ng/mL) incubation for 48 h in LX-2 cells. (A) Western blotting of phospho(p)-Smad 2, p-Smad 3, and Smad 2/3 in LX-2 cells. (B) Smad2/3 was measured by western blotting in LX-2 cells fractionated into the cytoplasm and the nucleus. PARP-1 was used for the nucleus marker. (C) LX-2 cells were stained with Smad 2/3 (red), nuclei (DAPI; blue), and merged (40× magnification; scale bar: 200 μm). (D) Western blotting of p-HER2, HER2, α-SMA, and β-actin in BT474 cells or activated hpHSCs.


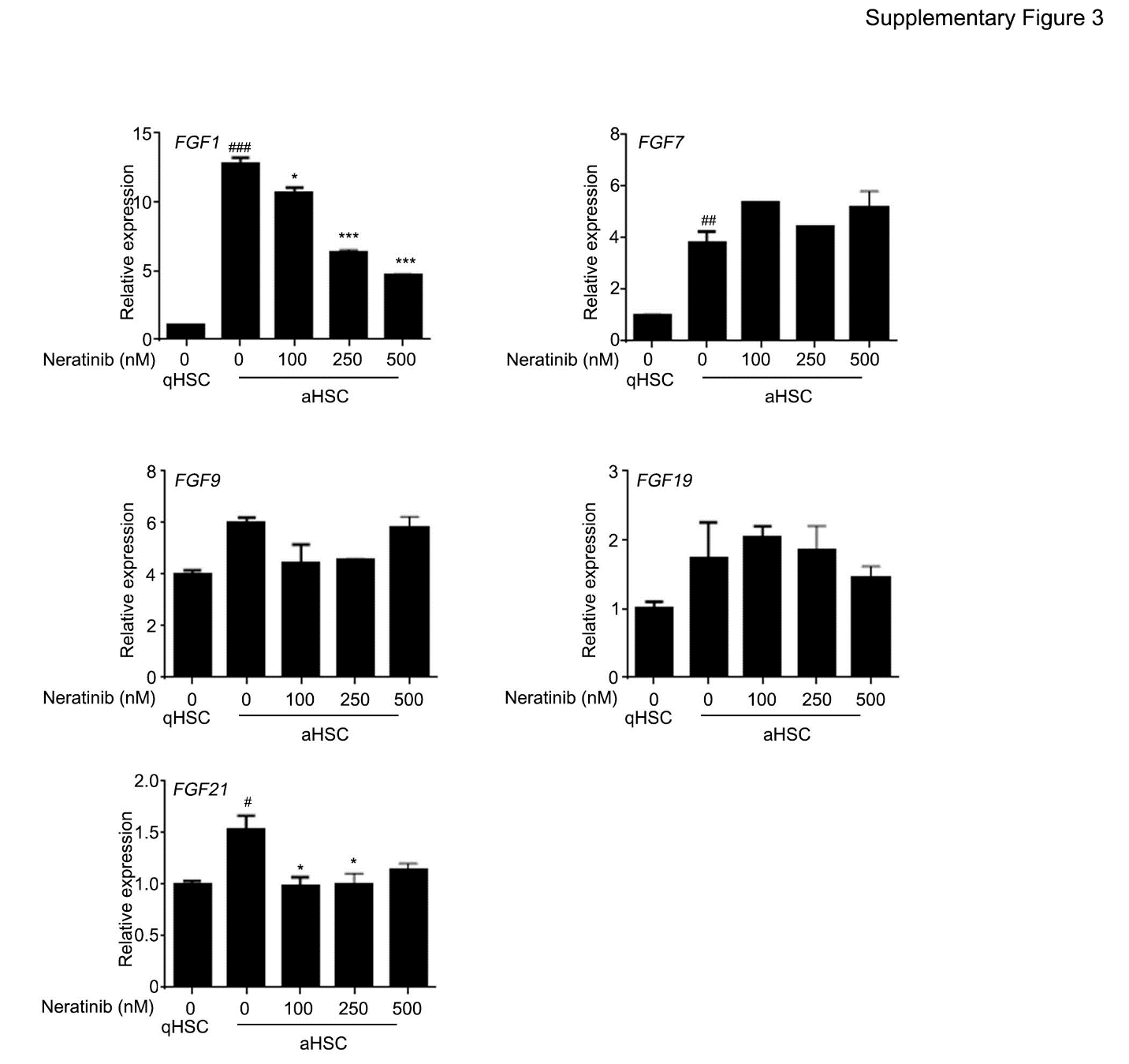


**Supplementary Figure 3.** Neratinib inhibits the expression of FGF1, and 2. Neratinib was treated in the activated hpHSCs as indicated, and qPCR performed using FGF1, 7, 9, 19, 21 specific primers.


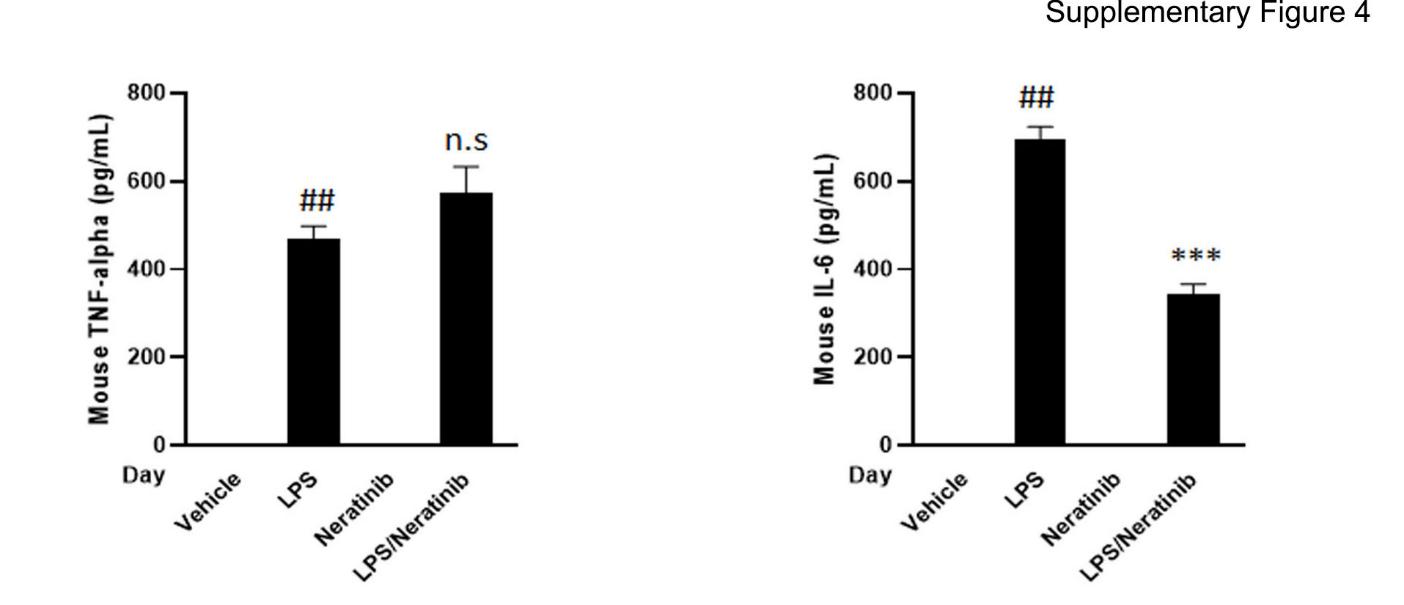


**Supplementary Figure 4**  Neratinib inhibits the IL-6 secretion n the LPS activated Kupffer cells. The level of mouse TNF-alpha and IL-6 determined after 4h of incubation with LPS (1 ug/mL) or/and neratinib (500 nM

Table.1 qPCR primer set for Human

| ***Gene*** | **Forward** | **Reverse** |
| --- | --- | --- |
| ***ACTA2*** | CCAGAGCCATTGTCACACAC | CAGCCAAGCACTGTCAGG |
| ***Col1A2*** | AGCAGGTCCTTGGAAACCTT | GAAAAGGAGTTGGACTTGGC |
| ***FGF1*** | ATGGCACAGTGGATGGGACAAG | TAAAAGCCCGTCGGTGTCCATG |
| ***FGF2*** | AGCGGCTGTACTGCAAAAACGG | CCTTTGATAGACACAACTCCTCTC |
| ***FGF7*** | CTGTCGAACACAGTGGTACCTG | CCAACTGCCACTGTCCTGATTTC |
| ***FGF9*** | CCAGGAAAGACCACAGCCGATT | CCATACAGCTCCCCCTTCTCAT |
| ***FGF19*** | TGCACAGCGTGCGGTACCTCT | CGGTACACATTGTAGCCATCTGG |
| ***FGF21*** | CTGCAGCTGAAAGCCTTGAAGC | GTATCCGTCCTCAAGAAGCAGC |
| ***Timp-1*** | GGAGAGTGTCTGCGGATACTTC | GCAGGTAGTGATGTGCAAGAGTC |
| ***GAPDH*** | AATCCCATCACCATCTTCCA | TGGACTCCACGACGTACTCA |

Table.2 qPCR primer set for mouse

| ***Gene*** | **Forward** | **Reverse** |
| --- | --- | --- |
| ***ACTA2*** | GTTCAGTGGTGCCTCTGTCA | ACTGGGACGACATGGAAAAG |
| ***Col1a2*** | CCGTGCTTCTCAGAACATCA | CTTGCCCCATTCATTTGTCT |
| ***Col3a1*** | GACCAAAAGGTGATGCTGGACAG | CAAGACCTCGTGCTCCAGTTAG |
| ***CCR2*** | GCTGTGTTTGCCTCTCTACCAG | CAAGTAGAGGCAGGATCAGGCT |
| ***Cox-2*** | GCGACATACTCAAGCAGGAGCA | AGTGGTAACCGCTCAGGTGTTG |
| ***FGF2*** | AAGCGGCTCTACTGCAAGAACG | CCTTGATAGACACAACTCCTCTC |
| ***IL-6*** | TACCACTTCACAAGTCGGAGGC | CTGCAAGTGCATCATCGTTGTTC |
| ***MCP-1*** | GCTACAAGAGGATCACCAGCAG | GTCTGGACCCATTCCTTCTTGG |
| ***PDGFR-b*** | TGGCCTCTGAGGACTAAAGC | AACAGAAGACAGCGAGGTGG |
| ***TNF-a*** | GGTGCCTATGTCTCAGCCTCTT | GCCATAGAACTGATGAGAGGGAG |
| ***TGF-b*** | TGACGTCACTGGAGTTGTACGG | GGTTCATGTCATGGATGGTGC |
| ***Timp-1*** | TCTTGGTTCCCTGGCGTACTCT | GTGAGTGTCACTCTCCAGTTTGC |
| **GAPDH** | TTG ATG GCA ACA ATC TCC AC | CGTCCC GTA GAC AAA ATG GT |


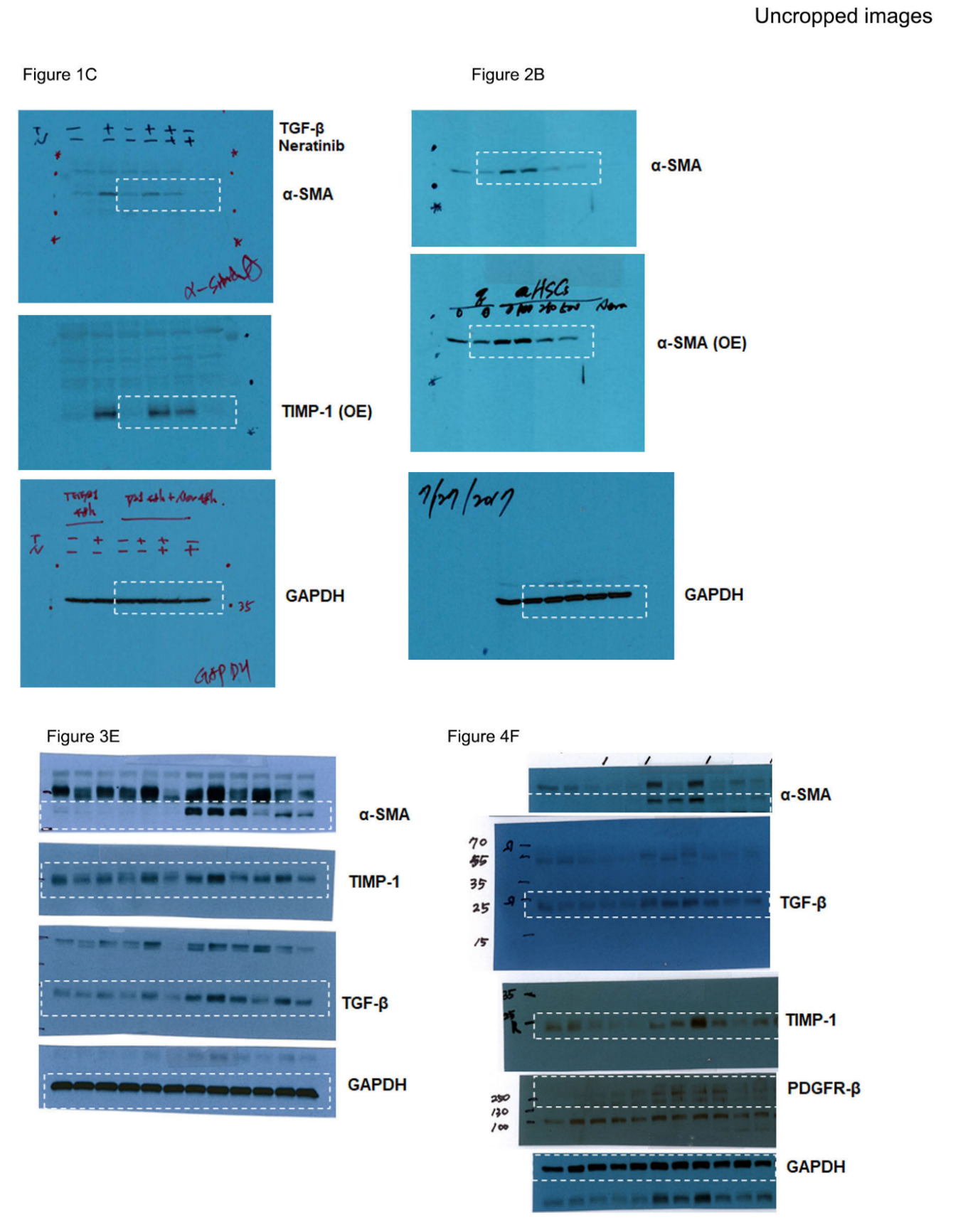


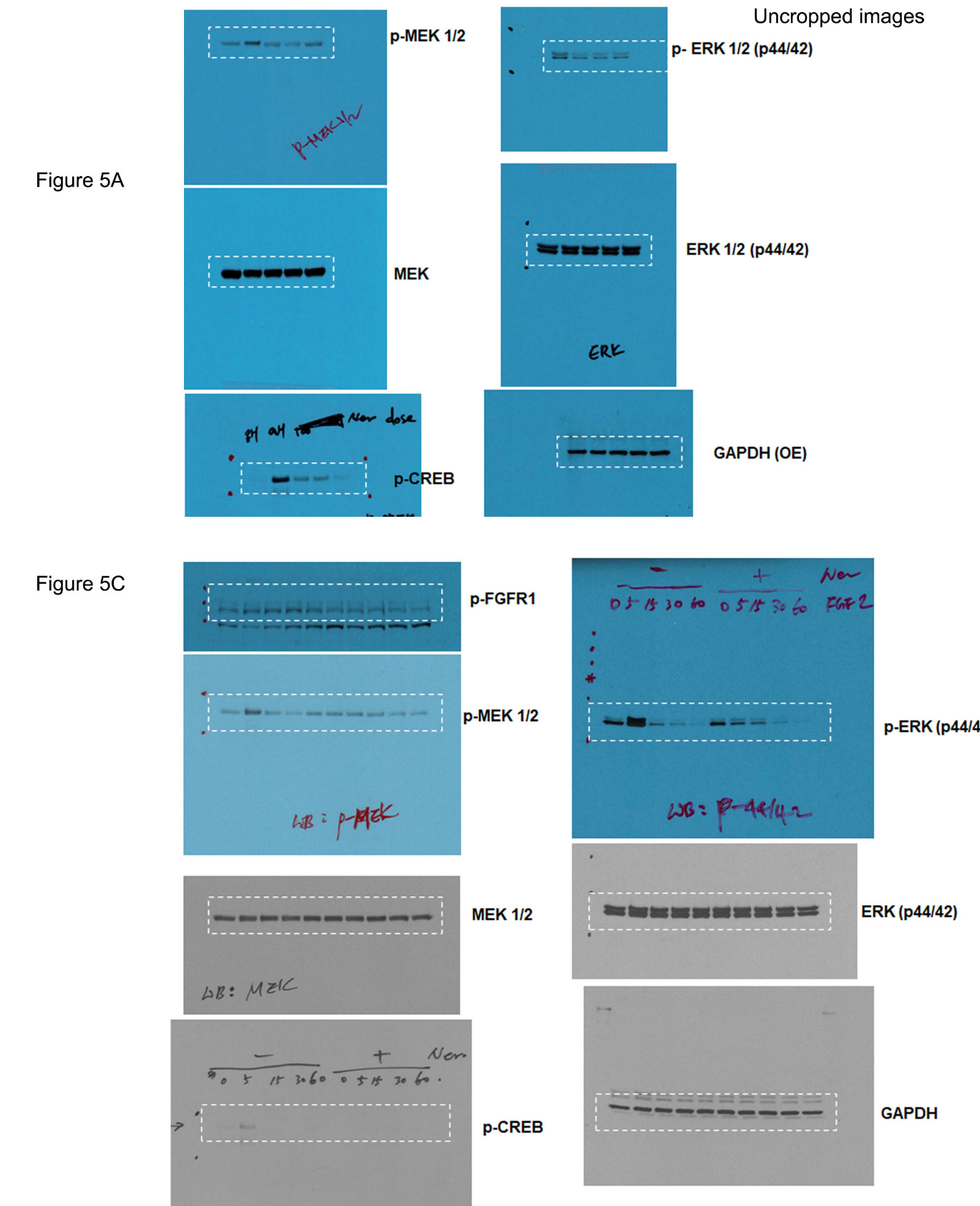


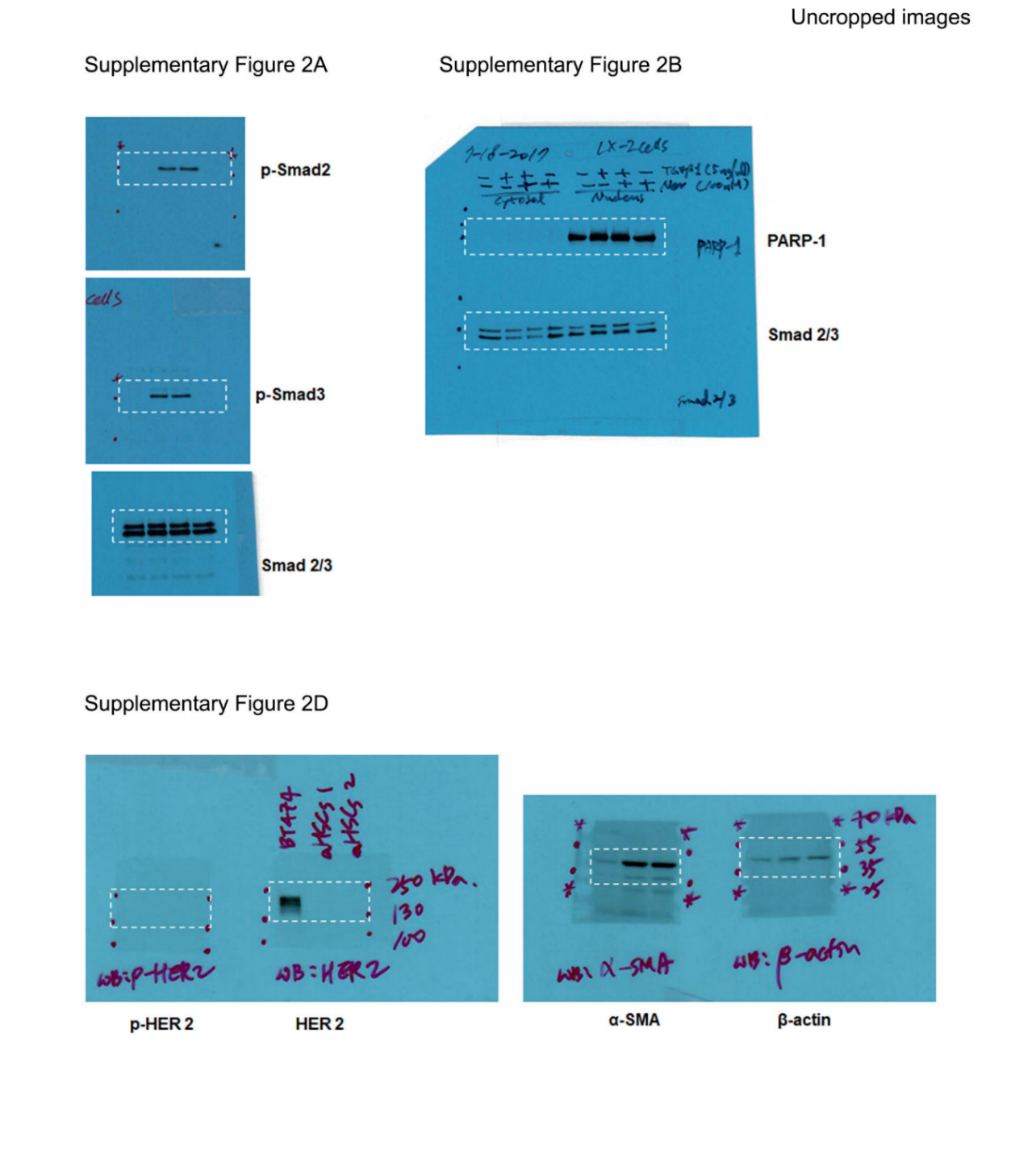

Supplement: Supplementary file 1 — Supplementary information [file 41598_2020_71688_MOESM1_ESM.docx]
